# Spatial Genetic Structure Patterns of Phenotype-Limited and Boundary-Limited Expanding Populations: A Simulation Study

**DOI:** 10.1371/journal.pone.0085778

**Published:** 2014-01-20

**Authors:** Qiang Dai, Xiangjiang Zhan, Bin Lu, Jinzhong Fu, Qian Wang, Dunwu Qi

**Affiliations:** 1 Chengdu Institute of Biology, Chinese Academy of Sciences, Chengdu, China; 2 Organisms and Environment Division, Cardiff School of Biosciences, Cardiff University, Cardiff, United Kingdom; 3 The Key Laboratory for Conservation Biology of Endangered Wildlife, Sichuan Province, Chengdu Research Base of Giant Panda Breeding, Chengdu, Sichuan, China; 4 Department of Integrative Biology, University of Guelph, Guelph, Ontario, Canada; University of Massachusetts, United States of America

## Abstract

Range expansions may create a unique spatial genetic pattern characterized by alternate genetically homogeneous domains and allele frequency clines. Previous attempts to model range expansions have mainly focused on the loss of genetic diversity during expansions. Using individual-based models, we examined spatial genetic patterns under two expansion scenarios, boundary-limited range expansions (BLRE) and phenotype-limited range expansions (PhLRE). Our simulation revealed that the genetic diversity within populations lost quickly during the range expansion, while the genetic difference accumulated between populations. Consequently, accompanying the expansions, the overall diversity featured a slow decrease. Specifically, during BLREs, high speed of boundary motion facilitated the maintenance of total genetic diversity and sharpened genetic clines. Very slight constraints on boundary motion of BLREs drastically narrowed the homogeneous domains and increased the allele frequency fluctuations from those levels exhibited by PhLREs. Even stronger constraints, however, surprisingly brought the width of homogeneous domains and the allele frequency fluctuations back to the normal levels of PhLREs. Furthermore, high migration rates maintained a higher total genetic diversity than low ones did during PhLREs. Whereas, the total genetic diversities during BLREs showed a contrary pattern: higher when migration was low than those when migration was high. Besides, the increase of migration rates helped maintain a greater number of homogeneous domains during PhLREs, but their effects on the number of homogeneous domains during BLREs were not monotonous. Previous studies have showed that the homogenous domains can merge to form a few broad domains as the expansion went on, leading to fewer homogeneous domains. Our simulations, meanwhile, revealed that the range expansions could also rebuild homogeneous domains from the clines during the range expansion. It is possible that that the number of homogeneous domains was determined by the interaction of merging and newly emerging homogeneous domains.

## Introduction

Species range expansion is an important biological process, which has shaped global biodiversity patterns. Species expand their ranges from natal sites at some point in their evolutionary history [1]. Global climatic changes have resulted in great changes in species distributions. Many Species have recolonized and/or expanded their ranges after the glaciation retreated [2], [3] or during the current rapid climate change caused by global warming [4], [5]. Long-distance introductions of alien species to new suitable areas produces another kind of species range expansions and are often harmful to native ecosystems [6]. Evolution in species' traits can also cause habitat adaptability change, and thus triggering a new round of range expansion [1].

The speed of expansions may vary considerably so that Nullmeier and Hallatschek [7] suggested two types of expansion, boundary-limited range expansion and phenotype-limited range expansion, which determine the speed of expansion by different mechanisms. A BLRE is typically a slow process, which happens when species expand their ranges along with the emergence of new, accessible, unoccupied, suitable habitat outside their current distribution areas. The rate at which suitable habitats become available is constrained by environmental changes over time. A classic example of the BLRE is that species expand their ranges from low-latitude to high-latitude regions in response to global warming [8], [9]. In addition to environmental changes, population expansion may also be limited by the Allee effect, characterized by reductions in the population growth in small or sparse populations [10] and slower rate of range expansions [11], [12]. In PhLREs, however, the speed of expansion is determined primarily by population's intrinsic characteristics, e.g. reproduction rate, migration rate and distance. When PhLREs happen, a species can claim a vast unoccupied suitable habitat once it becomes accessible when the Allee effect is either weak or absent. The successful invasion of an invasive species into a new continent likely represents a PhLRE scenario [13]–[15]. Moreover, a PhLRE may also occur when a new beneficial mutation expands a species' adaptive zone, hence changing the definition of its suitable habitat [16].

Several previous studies have investigated the spatial genetic consequences of PhLREs. In general, a species' range expansion is often accompanied by loss of genetic diversity because of the founder effect [2], [17]–[19]. However, this loss is not uniform, which recently has caught much attention. For example, both petri dish experiments using microbial species [20], [21] and model simulations [11], [20]–[22] revealed a pattern characterized by the emergence of genetically homogeneous domains from genetically mixed populations, which then merged during expansion. Hallatschek and Nelson [11] suggested that this pattern was driven by strong genetic drift at the fronts of expanding populations. Meanwhile, allele frequency gradients were formed between genetically homogeneous domains as a consequence of migration leading to allele frequency clines orthogonal to the expansion direction [23].

The spatial genetic consequences of BLREs, although as important as those of PhLREs, have rarely been investigated, with the exception of simulations carried out by Nullmeier and Hallatschek [7], in which BLREs were found to maintain a higher level of genetic diversity than PhLREs were. Hallatschek and Nelson [11] and Roques et al. [12] also studied the genetic consequences of BLRE-like expansions caused by the Allee effect, and concluded that it also increased genetic diversity at expansion fronts.

In this study, we aim to explore different spatial patterns of genetic variations, specifically the number of genetically homogeneous domains and the fluctuations of allele frequencies, at the expansion front generated by the PhLREs and BLREs with varied speeds of boundary motion. We simulated the population genetic dynamics of neutrally evolved alleles at the advancing fronts using individual-based models (IBM). The impacts of migration and reproduction on the spatial genetic patterns were also examined.

## Methods

### (a) Model description

The simulations were carried out using a lattice with 75 row ×200 column grids. The populations comprised asexual, haploid individuals with discrete generations. Each individual genotype was typed by one neutral diallelic locus. We defined three population parameters: carrying capacity (*K*), reproduction rate (*r*) and migration rate (*m*). As noted by Excoffier and Ray (2008), small populations tend to produce less defined homogeneous domains and broader transition zones, therefore we set *K* to 100 in our simulations, which was much greater than that used in Korolev et al.'s (2011) simulations (*K* = 30), and was within the range suggested by Excoffier and Ray's (2008) simulations. For reproduction, the number of offspring of each individual was drawn from independent Poisson distributions with means of *r* = 1.1, 2, 3, 5. Individuals were assumed to die immediately after reproduction. The migration rate *m* were set to 0.01, 0.1, 0.2, and 0.4, which lied within the range employed in the previous simulations [23], [24]. To examine the effect of reproduction rate, varied *r* values (*r* = 1.1, 2, 3, 5) were tested with a constant *m* of 0.2. To test the effect of migration rate, different *m* values (*m* = 0.01, 0.1, 0.2, 0.4) were used with a constant *r* of 3. The individuals migrated with a probability of 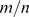 to any of the immediately adjacent *n* demes. The value of *n* varied from 2 to 4, depending on the position of each deme with respect to the lattice boundaries.

Each simulation started with populations in the first 20 deme columns at the far left of the lattice; all the those populations had size of *K* and allele frequency of 0.5 for each allele. We also simulated with initial allele frequencies of 0.01, 0.1 and 0.2 with a constant *m* of 0.2 and *r* of 3 to test the impact of initial frequency.

As the simulations progressed, the species range expanded from left to right of the lattice. The order of events in each generation was reproduction, migration, and finally random removal of individuals in demes where population size exceeded *K*. Each simulation was run for 8,000 generations. When a column of previously unoccupied grids reached *K*, the genetic components of those demes were recorded as a frozen record.

### (b) Boundary-limited and phenotype-limited range expansion models

Two range expansion scenarios, BLRE and PhLRE, were simulated. During PhLRE, all grids were designated as suitable habitats and range expansion occurred immediately after the simulation was initialized. During BLRE scenarios, only the first 20 columns were designated as suitable habitats at the initialization, while the others were all unsuitable habitat. The individuals can not reproduce (*r* = 0) in the unsuitable habitat. The unsuitable grids gradually turned into suitable, column by column, with the progression of generations. This process was used to imitate habitat expansion driven by environmental change. We employed six different speeds of boundary motion in BLREs, taking 600, 1000, 2000, 4000, 6000 and 7900 generations for all of the grids to change into suitable habitats. Other parameters were set as follows: *r* = 3, *m* = 0.2, and the initial allele frequency  = 0.5. Our simulation pilots showed that it took a mean of 533.6 generations for populations to expand from left to right and all populations reach *K* during a PhLRE, given *r* = 3 and *m* = 0.2. Therefore, 600 generations seemed to be a slight constraint on expanding populations. Additionally, the populations that do not expand make the extreme BLRE, which is usually referred as a process of isolation by distance [25]–[27]. We also carried out simulations with stationary habitats for non-expanded populations: only the first 20 columns were designated as suitable habitats, and the boundary was assumed to have never moved. The populations in the 20th column were considered as a “stationary front” and were compared with populations at expansion fronts during BLREs and PhLREs.

### (c) Model analysis

We counted the homogeneous domains and clines and calculated their mean widths at expansion fronts. A genetically homogeneous domain was defined as one or multiple spatially continuous homogeneous populations, and an allele frequency cline was defined as one or multiple spatially continuous genetically mixed populations.

Subtle allele frequency fluctuations orthogonal to the expansion direction were analyzed by Fast Fourier Transform (FFT) algorithm, which has been widely used in diverse fields of science to describe periodic patterns. The allele frequencies of a column of demes were considered as a wave curve *p*, and the mean frequency of the power spectrum (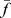) was calculated: 
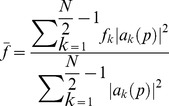
(1)


Where 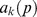 is the FFT of curve *p* at index *k*, and 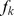 is the frequency corresponding to *k*. The term of 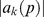 is the amplitude at 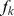. *N* is the number of demes in a column, and the highest frequency can be identified as 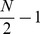 since *N* is odd in our simulations. A higher 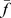 indicates more fluctuation of allele frequency. More homogeneous domains will also increase the 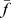.

Genetic differentiation within and among populations at an expansion front was assessed by the average number of pairwise differences within populations (PX), the average number of pairwise differences between populations (PXY), and the corrected average pairwise difference between populations (Pc) [28], [29]. Pc was calculated as 
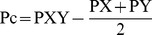
(2)


Each parameter combination was simulated for 100 times. The counts and width of genetically homogeneous domain and clines, 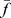, and pairwise differences were computed for each simulation, and then averaged for each parameter combination. This modeling was implemented in Java, and all the other analyses were performed in R 2.15.2 [30].

## Results

### (a) Genetically homogenous domains and clines

**Figure 1:**
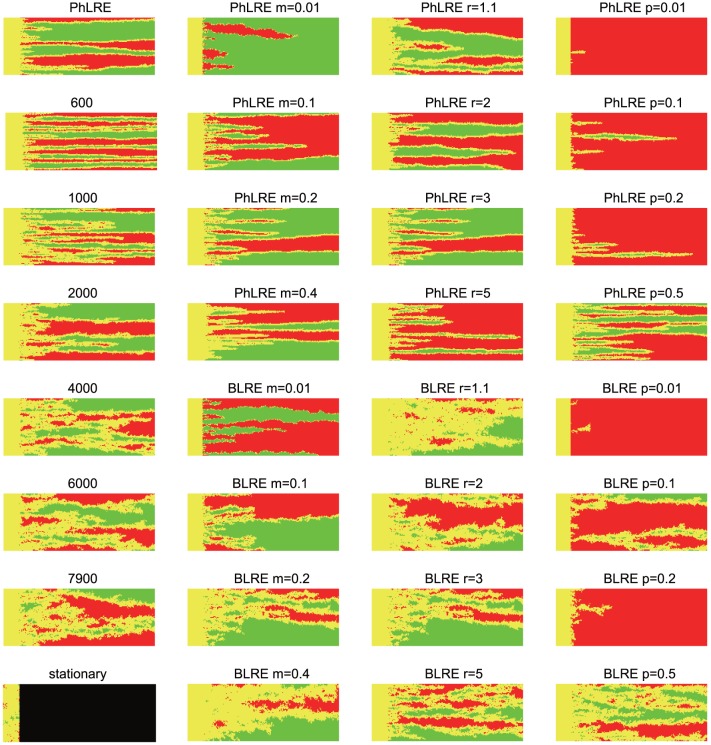
Spatial distribution of allele frequencies in the expanding populations. The allele frequencies were recorded when all populations in a column first reached their carrying capacity. The first column showed expansions with varying speeds of boundary motion (the number of generations to complete a motion), while the other parameters remained constant (*r* = 3, *m* = 0.2, initial frequency  = 0.5) in the first column. The ‘stationary’ referred to the non-expansion simulations carried out in stationary habitats, and the snapshots of genetic compositions of populations were recorded at generation 8000. The second to fourth columns showed expansions with varying migration rates, reproduction rates and initial frequencies, respectively. Column 2: *r* = 3, time to complete a boundary motion was 6000 generations, initial frequency  = 0.5; Column 3: *m* = 0.2, time to complete a boundary motion was 6000 generations, initial frequency  = 0.5; Column 4: *r* = 3, *m* = 0.2, time to complete a boundary motion was also set to 6000 generations. Carrying capacity was 100 in all the simulations. Green indicated an allele frequency of 0, red an frequency of 1, yellow an intermediate frequency, and black denoted unoccupied grids.

**Figure 2:**
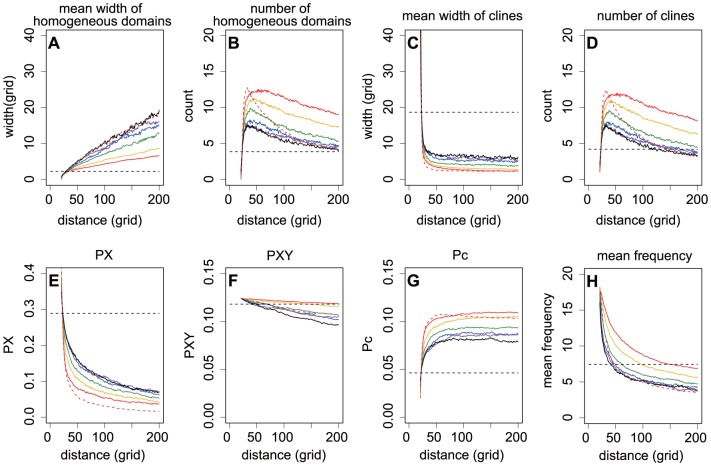
The spatial patterns of genetic diversity at expansion fronts given various boundary motion speeds. The spatial patterns of genetic diversity were described by the width and number of genetically homogeneous domains (A, B) and genetic clines (C, D), pairwise differences in PX (E), PXY (F) and Pc (G), and the mean frequency of the power spectrum (H) at expansion fronts during PhLREs (red dashed lines) and BLREs (solid lines). The speeds of boundary motion, indicated by generation to complete the boundary motion, varied from 800 to 7900 generations (red: 800, yellow: 1000, green: 2000, blue: 4000, purple: 6000, dark: 7900). The black dashed lines described the spatial genetic patterns at a distance of 20 grids and 8000 generations, which were generated in the non-expansion simulations. Other parameters were set as follows: *r* = 3, *m* = 0.2, and the initial allele frequency  = 0.5.

For both the PhLRE and BLRE, many narrow homogeneous domains emerged very quickly at the beginning of the expansions (Fig. 1). Correspondingly, the number of both genetically homogeneous domains and clines increased suddenly. The homogeneous domain expanded at this stage, coupled with the quick narrowing of clines (Fig. 2). As the expansion went on, the number of homogeneous domains and clines decreased while some narrow homogenous domains merged to form a few broad domains, although the average width of the clines remained stable after the initial narrowing. However, the subtle allele frequency fluctuations 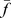 increased much earlier than number of homogeneous domains did, and kept decreasing during the expansion.

The PhLREs exhibited the narrowest clines, however slow boundary motion of BLREs broadened the clines (Fig. 2). In the non-expanded populations, genetically homogeneous domains and clines were formed in the stationary habitats, possibly due to the effect of isolation by distance [25]–[27]. As shown by Figure 2, the width of clines at the 20th column after 8000 generations was much wider than those at expansion fronts during BLREs or PhLREs. Additionally, the more slowly the boundary expanded, the broader the homogeneous domains were. Similarly, the slower the boundary expansions were, the higher 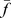 values. Altough PhLREs can be considered as the fastest boundary motion and the non-expansions as the slowest, the width of homogeneous domains was very broad in the former, but narrowest in the latter. Besides, the 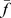 values were the lowest during PhLREs and the highest during non-expansion process.

**Figure 3:**
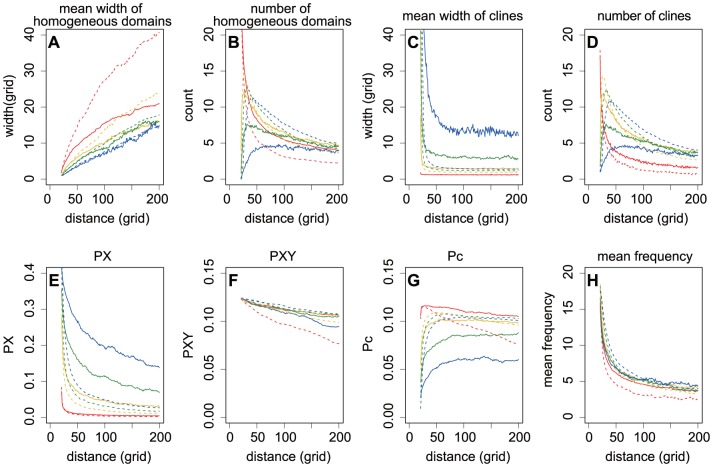
The effects of migration rates on spatial patterns of genetic diversity at expansion fronts. The migration rates varied from 0.001 to 0.4 (red: 0.01, yellow: 0.1, green: 0.2, blue: 0.4). The spatial patterns of genetic diversity were described by width and number of genetically homogeneous domains (A, B) and genetic clines (C, D), pairwise differences in PX (E), PXY (F) and Pc (G), and the mean frequency of the power spectrum (H)). The solid and dashed lines indicated BLRE and PhLRE, respectively. The value of *r* was set as 3; initial allele frequency  = 0.5; and the number of generations to complete a boundary motion equal to 6000.

**Figure 4:**
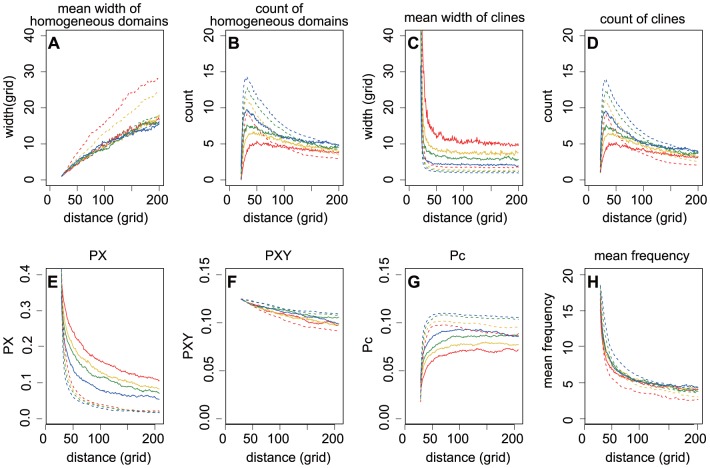
The effects of reproduction rates on spatial patterns of genetic diversity at expansion front. The spatial patterns of genetic diversity described by the width and number of genetically homogeneous domains (A, B) and genetic clines (C, D), pairwise differences in PX (E), PXY (F) and Pc (G), and the mean frequency of the power spectrum (H). The solid and dashed lines indicated BLREs and PhLREs, separately. The reproduction rates varied from 1.1 to 4 (red: 1.1, yellow: 2, green: 3, blue: 4). The value of *m* was set as 0.2; initial allele frequency as 0.5; and the number of generations to complete a boundary motion 6000.

High migration rates broadened the clines but narrowed the homogeneous domains (Fig. 3). It also increased the 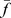, especially during PhLREs. In contrast, low migration rates increased the number of homogeneous domains more sharply than did high migrations rates at the early stage of the expansion. In the end, the higher migration rates helped maintain a greater number of homogeneous domains during PhLREs than lower ones did. However, the effect of increasing migration rate on the domain number was not monotonous during BLREs. The high reproduction rate maintained high number of homogeneous domains and clines, narrowed the clines, but broadened the homogeneous domains at least during PhLREs. The high reproduction rate also increased the 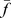, especially during PhLREs (Fig. 4).

**Figure 5:**
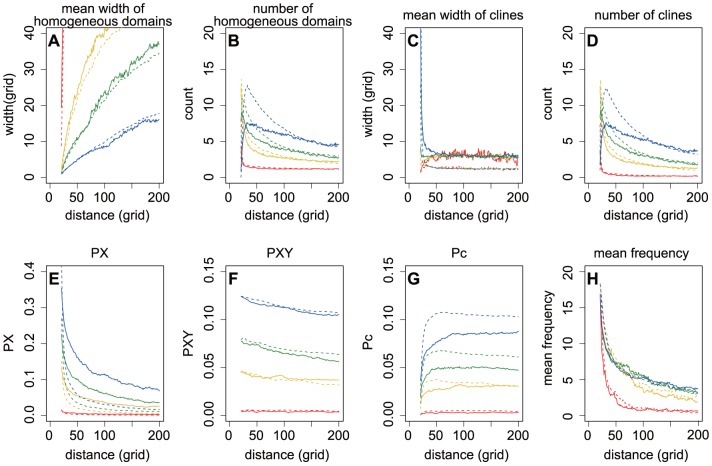
The spatial patterns of genetic diversity at expansion fronts when initial allele frequencies varied. The values of initial allele frequencies varied from 0.01 to 0.5 (red: 0.01, yellow: 0.1, green: 0.2, blue: 0.5). The solid indicated BLREs and dashed lines represented PhLREs. A to D showed the width and number of genetically homogeneous domains and genetic clines. E to F showed the pairwise differences in PX, PXY, and PC respectively. H showed the mean frequency of the power spectrum. The value of *r* was set to 3; *m* to 0.2; and the number of generations to complete a boundary motion 6000.

Initial allele frequencies deviated from 0.5 accelerated the fixation and led to fewer but broader homogeneous domains and narrower clines (Fig. 5), because populations with skewed allele frequencies were more likely to lose rare alleles during the random drift and facilitated the emergence and merging of homogeneous domains. The initial skewed allele frequencies also decreased the 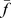.

### (b) Population genetic heterogeneity

The simulations revealed that at the beginning of an expansion, pairwise differences within populations (PX) decreased quickly at the advancing front, while the corrected average pairwise difference between populations (Pc) increased quickly. The changing rate of PX and Pc soon decreased with the expansion (Fig. 2, 3, 4, 5). The more slowly the boundary changed, the higher the PX and lower the Pc was. And the highest PX and lowest Pc occurred in the simulations with non-expanded populations (Fig. 2). Pc during PhLREs peaked at the early stage of expansion and then declined slightly in the long run. This was because most populations at the expansion fronts became genetically homogeneous during PhLREs and thus exhibited low Pc values. The average pairwise difference between populations (PXY) decreased slowly with expansion. The higher speed of boundary motion generally maintained higher PXY than the lower speed did. However, PhLREs and simulations with stationary habitats demonstrated the exceptions again: PXY was intermediate during the former, but very high during the latter.

Generally, high migration rate increased the PX, but decreased the Pc (Fig. 3). However, there was one exception in that the Pc dropped quickly when migration rate was very low during a PhLRE. This was because most of the populations at the expansion front became genetically homogeneous under this scenario. High migration rate increased the PXY during PhLREs, but decreased it during BLREs with slow speeds of boundary motion. Reproduction rate decreased the mean genetic heterogeneity within populations, but increased Pc and PXY (Fig. 4). Initial allele frequencies deviated from 0.5 led to lower values of PX, PXY or Pc (Fig. 5).

## Discussion

### (a) The difference between PhLREs and BLREs

Our simulations showed stripe-like genetic patterns in expanded populations, which were equivalent to the genetic sectoring patterns established by previous studies [20], [21], [23]. Slow boundary motion, which had a strong limitation on expansion, broadened the genetic clines between homogeneous domains. PhLREs, the fast expansion, exhibited the narrowest clines and non-expansion simulations exhibited the broadest clines. This is not surprising because the expansion fronts halted for several generations before the next expansion and more migration occurred between populations at the expansion fronts during slow expansions. Similarly, Hallatschek and Nelson [11] and Roques et al. [12] also reported increased genetic diversities at expansion fronts under slow range expansions caused by the Allee effect.

The speed of boundary motion exerts great impacts on the spatial genetic pattern of the advancing front. For example, very slight constraints on boundary motion could drastically narrowed the homogeneous domains and increased the allele frequency fluctuations from those levels exhibited by PhLREs (Fig 2). In contrast, the homogeneous domains were broadened and allele frequency fluctuation decreased with even slower boundary motion. However, the strong constraint on boundary motion brought those values back to the levels exhibited by PhLREs. Simulations with non-expanded populations, though considered as the slowest boundary motion, behaved differently in that the narrowest homogenous domains were formed and the highest level of allele frequency fluctuation reached. This maybe because the founder effect was totally absent in these simulations of stationary habitats, and it was the migration-drift balance has resulted in this spatial genetics patterns of genetic [25]–[27]. As for BLREs, the founder effect, however, always existed once the boundary began to expand, no matter how slow it progressed.

The average number of pairwise differences revealed a clear picture of changing spatial genetic patterns during expansions. The slow decrease in PXY, fast loss of PX, and fast increase in Pc at the expansion front suggested a process represented by slow loss of total diversity, quick loss of that within populations, and quick accumulation of genetic heterogeneity between populations corresponding to the emergence and merging of genetically homogenous domains.

The speed of boundary motion also influenced the genetic diversity at advancing fronts. Slow boundary motion increased the diversity within populations but decreased the heterogeneity between populations. Though Nullmeier and Hallatschek [7] suggested that the total loss of diversity did not depend on the speed of the range expansion, our results revealed that at least high speed of boundary motion could facilitate the maintenance of total genetic diversity during BLREs through quickly accumulating genetic heterogeneity between populations. This discrepancy maybe result from different model conditions: Nullmeier and Hallatschek [7] assumed that the front had expanded so slowly that the population density stayed at constant density up to the front. In our model, this condition could only be satisfied if the boundary motion was sufficiently slow.

Under PhLRE, though species ranges expanded most quickly, the total genetic diversity was intermediate among those during BLREs. For simulations with stationary habitats, the absence of founder effect maintained the total genetic diversity at the “non-expanding fronts”. These results indicated that PhLRE and non-expansion simulations can not be simply considered as BLREs with the quickest or the slowest boundary motions.

Some previous empirical studies have implied differences in genetic patterns between BLRE and PhLRE populations. For example, examinations of several invasive species have revealed a decrease in genetic variation at expansion fronts [31], [32]. However, the European larch (*Larix decidua*) maintained high genetic diversity during slow range expansion due to global warming, which has usually been attributed to the intensive mixing of genes [2]. Nevertheless, homogenous sectoring or stripe patterns are not easy to find in natural populations [32]–[35] because such patterns are difficult to reveal without intensive sampling. Nullmeier and Hallatschek [7] suggested that the lineage has to be sampled from a distance of more than a length *L*_plateauto_  =  2*Km* to detect a range expansion, where *K* was the carrying capacity and *m* was the migration rate. Furthermore, many factors, e.g. landscape heterogeneity and multiple introductions [36], can further complicate patterns of spatial genetic structure.

It is notable that spatial expansions, can not only lead to allele frequency clines by eroding genetically homogeneous domains, but also rebuild genetically homogeneous domains from those clines by the ongoing demixing (Fig. 1). It is possible that the number of homogeneous domains in our study is the consequence of emergence and merging of homogeneous domains. The emergence of homogeneous domains could explain the fluctuation in the average numbers of genetically homogeneous domains during BLREs. However, the emergence of new homogeneous domains was rare, if any, during PhLREs. Therefore, smaller fluctuation in the average numbers of homogeneous domains was observed in PhLRES than that in BLREs (Fig 2, 3, 4, 5). Previous models [20], [37], [38] have shown the merging of homogeneous domains together with expansion, but failed to detect the emergence of new homogeneous domains from clines. The pattern of new homogeneous domains during BLREs was similar to those created by mutations and selection at expansion fronts [39]. However, their underlying mechanisms were totally different: the emergence of new homogeneous domains during BLREs was driven by the interaction of migration and demixing processes, as shown in our study, which differs from Kluver [40] ' s model, where the new stripes emerged from mutations.

### (b) The effects of migration rate and reproduction rate

High migration enhanced genetic diversity within populations but reduced heterogeneity between populations. Surprisingly, we found that high migration rate maintained a higher total genetic diversity than low migration rate during PhLREs, but the situation was reversed during BLREs, though Nullmeier and Hallatschek [7] suggested that the larger the migration rate, the smaller the loss of diversity. This is because high migration rates could increase both speed of range expansion and gene flow during PhLREs, but exerted little effect expansion speed during BLREs. The non-monotonous effect of increasing migration rate on the number of homogeneous domains during BLREs was also somehow counterintuitive. Specifically, the number of homogeneous domains was determined by the equilibrium of merging and emergence of homogeneous domains. High gene flow broadened the genetic clines and allowed more homogeneous domains to emerge, however, it quickly eroded existing homogeneous domains [23]. Low migration however failed to maintain broad clines and thus new homogeneous domains rarely emerge from the clines (Fig. 1,3).

Our simulations suggested that high reproduction rate reduced the genetic diversity within populations but increased the genetic heterogeneity between populations. It also narrowed the clines. It is possible that the high *r* diluted effects of migration by reproducing more individuals from local population, and thus hindered the formation of clines. This was exactly the case in BLREs, where front populations had more time to reproduce and migrate. High reproduction also increased the number of homogeneous domains and clines during PhLREs, but not so drastically during BLREs (Fig 3). Possibly, this is because the high *r* has another effect, i.e. lessening drift and demixing by quickly increasing population size, which will in turn impede the formation and merging of homogeneous domains. During PhLREs, species ranges kept expanding before the front populations had reached *K*, and a high *r* helped those populations to reach larger sizes and lessen drift. During BLREs, however, front populations had enough time to obtain large sizes before further expansion took place, therefore, a high *r* value could not guarantee a noticeable effect.

### (c) Methodological considerations

The Fast Fourier Transform (FFT) may prove to be a powerful tool for the detection of spatial genetic structure. In evolutionary and ecological studies, the period of recurrent spatial pattern was often approximately evaluated by spatial autocorrelation analysis [41]. Korolev et al. [22] presented the genetic sectorial structures by plotting the spatial heterozygosis as a function of angle, which was equivalent to a spatial autocorrelation analysis. Evaluating the allele frequency fluctuations by spatial autocorrelation analysis, nevertheless, is not realistic when the periods of fluctuation are highly variable. The spatial autocorrelation index is evaluated based on an averaged correlationship among all sites, by which the stochastic information of stripes is lost. In contrast, the FFT analysis can reveal more subtle fluctuations of allele frequency. Though the number of genetically homogeneous domains exhibited a fast increase at the early stage of expansion, 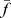 increased much earlier and showed an emergency of genetic fluctuations before the formation of homogeneous domains.
