# Correction: Spatial Genetic Structure Patterns of Phenotype-Limited and Boundary-Limited Expanding Populations: A Simulation Study

**DOI:** 10.1371/annotation/92a1ff96-eb6f-45f8-bc66-09c08e0dbeeb

**Published:** 2014-01-30

**Authors:** Qiang Dai, Xiangjiang Zhan, Bin Lu, Jinzhong Fu, Qian Wang, Dunwu Qi

A funding organization and grant to the last author (DQ) are incorrectly omitted from the Funding Statement. The Funding Statement should read: "This project is supported by a National Natural Science Foundation of China grant and a National Sciences and Engineering Research Council Discovery grant to JF, Leading Foundation of the Chinese Academy of Sciences (XDA05050404) to QW and Sichuan Science and Technology Department Program (2012SZ0094) for DQ. The funders had no role in study design, data collection and analysis, decision to publish, or preparation of the manuscript."

